# Determinants of long-term survival in patients with IDH-mutant gliomas

**DOI:** 10.1007/s11060-024-04826-9

**Published:** 2024-09-24

**Authors:** Sophie Katzendobler, Sebastian Niedermeyer, Jens Blobner, Christoph Trumm, Patrick N. Harter, Louisa von Baumgarten, Veit M. Stoecklein, Joerg-Christian Tonn, Michael Weller, Niklas Thon, Jonathan Weller

**Affiliations:** 1 Department of Neurosurgery, LMU University Hospital, LMU Munich, Marchioninistrasse 15, 81377 Munich, Germany; 2 Department of Neuroradiology, LMU University Hospital, LMU Munich, Germany; 3 Center for Neuropathology and Prion Research, LMU University Hospital, LMU Munich, Munich, Germany; 4 German Consortium for Translational Cancer Research (DKTK), Partner Site Munich, Heidelberg, Germany; 5 Department of Neurology, University Hospital and University of Zurich, Zurich, Switzerland

**Keywords:** Astrocytoma, Lower grade glioma, Chemotherapy, IDH-mutation, Oligodendroglioma, Radiotherapy, Temozolomide

## Abstract

**Background:**

Survival times of patients with IDH-mutant gliomas are variable and can extend to decades. Many studies provide progression-free rather than overall survival times and prognostic factors remain ill-defined. Here we explored characteristics of short- and long-term survivors within a cohort of patients with extended follow-up.

**Methods:**

This single-center, case-control study included 86 patients diagnosed between 1998 and 2023 who either died within 6 years after diagnosis or survived at least 15 years. Patient characteristics and prognostic factors were stratified by short- (< 6 years) versus long-term (≥ 15 years) survival.

**Results:**

Forty-seven patients (55%) diagnosed with astrocytoma and 39 patients (45%) with oligodendroglioma were included retrospectively. Median follow-up of the survivors was 16.6 years (range 15-28.9). Thirty-four deaths (40%) had been reported at database closure. Long-term survival was associated with CNS WHO grade 2 (*p* < 0.01), smaller tumor volumes (*p* = 0.01), lack of contrast enhancement (*p* < 0.01), wait-and-scan strategies (*p* < 0.01) and female sex (*p* = 0.04). In multivariate analyses for oligodendroglioma, larger T2 tumor volumes were associated with shorter survival (HR 1.02; 95% CI 1.01–1.05; *p* = 0.04). In patients with astrocytoma, lack of contrast enhancement (HR 0.38; 95% CI 0.15–0.94; *p* = 0.04) and wait-and-scan strategies (HR 5.75; 95% CI 1.66–26.61; *p* = 0.01) were associated with longer survival.

**Conclusion:**

Large T2 tumor volume and contrast enhancement may be important risk factors for shorter survival, while age might be of lesser importance. Wait-and-scan strategies may yield excellent long-term survival in some patients with astrocytoma.

**Supplementary Information:**

The online version contains supplementary material available at 10.1007/s11060-024-04826-9.

## Introduction

Gliomas with mutations in the isocitrate dehydrogenase (IDH) genes 1 or 2 often affect young to middle-aged adults [1]. Beyond the frequent manifestation by an epileptic seizure, patients are typically asymptomatic or oligosymptomatic [2, 3]. The prognosis of patients with IDH-mutant glioma is highly variable. Some patients die within months whereas others may survive for decades [4]. Due to the typically long survival times of patients with CNS WHO grade 2 gliomas and in contrast to CNS WHO grade 3 astrocytomas, prospective studies might not reach median survival times [5–7]. In CNS WHO grade 3 astrocytomas, the CATNON trial has reported a median overall survival (OS) of 8.1 years for grade 3 and 4.7 years for grade 4 astrocytomas. Several risk factors for poor OS have been described, but some of these remain controversial. Higher age, neurological deficits, and larger tumor burden at diagnosis as well as larger postoperative residual tumor volume seem to be associated with shorter progression-free survival (PFS) and OS in patients with WHO grade 2 and 3 gliomas [8–12]. Initial contrast enhancement has been described as a poor prognostic factor, too [13]. Higher WHO grade as an independent risk factor remains debated because of high interrater variability in histological grading, divergent study results and a treatment bias [4, 14]. Recent studies have highlighted the prognostic implications of homozygous deletions of the cyclin-dependent kinase inhibitor 2 A or B (CDKN2A/B) since patients with such tumors show especially poor outcome [15, 16]. These findings have been implemented in the 2021 WHO classification. IDH-mutant astrocytomas are classified as CNS WHO grade 4 if a CDKN2A/B homozygous deletion is detected, irrespective of histological features such as necrosis or microvascular proliferation [17]. CDKN2A/B analysis has, however, not yet been implemented in daily clinical routine in most centers.

In summary, there is no clear consensus on risk factor assessment and weighting in patients with IDH-mutant gliomas and thus, treatment recommendations may vary substantially between centers. Evidence-based guidance is provided by the current EANO and ASCO-SNO guidelines on the diagnosis and treatment of diffuse gliomas of adulthood that acknowledge age, neurological status, and postoperative residual tumor volume as independent risk factors and gives leeway for wait-and-scan strategies in younger patients and after gross total resection [14, 18]. It is unclear whether wait-and-scan strategies, i.e., leaving the tumor to its natural evolution, can jeopardize long-term outcome. Early versus delayed radiotherapy was not associated with survival differences in patients with WHO grade 2 gliomas, admittedly in a now historical study [19]. Tumor-specific therapy options comprise sequential radiochemotherapy with procarbazine, CCNU (lomustine) and vincristine (PCV) or, less evidence-based, temozolomide. These regimens have yielded excellent long-term tumor control rates, but the corresponding clinical trials oftentimes recruited patients with WHO grade 3 gliomas or were conducted before the implementation of the IDH status as the major stratification factor for gliomas of adulthood [5, 8, 20–24]. It also remains to be determined whether tumor-specific therapy can push some gliomas towards a more aggressive evolution through selective pressure. A few studies indicate induction of hypermutation associated with inferior OS in some patients treated with temozolomide, although this has not been validated in prospective trials, and clinical implications are yet to be determined [25–28].

With the present scenario of potentially toxic and exhausting treatment regimens for relatively young, oftentimes asymptomatic patients who might survive for decades, multidimensional risk factors analysis may provide important guidance to determine time point, necessity, and modality of tumor-specific therapy. Here we compare a large cohort of long-term survivors with an OS of 15 years or longer after diagnosis of an IDH-mutant oligodendroglioma or astrocytoma with a reference group of patients with less favourable outcome to determine prognostic factors for long-term survival. Based on the results of previous studies, we set a cutoff of 6 years for short-term survival in patients with IDH-mutant gliomas [5–7]. Within the framework of preexisting literature on risk factors for short OS, we hypothesize that tumor size and contrast enhancement on MRI might be important markers for prognosis. Furthermore, we hypothesize that patient age at diagnosis might be a less important risk factor for patients with IDH-mutant as opposed to IDH-wildtype gliomas.

## Patients and methods

### Patient evaluation

**Fig. 1:**
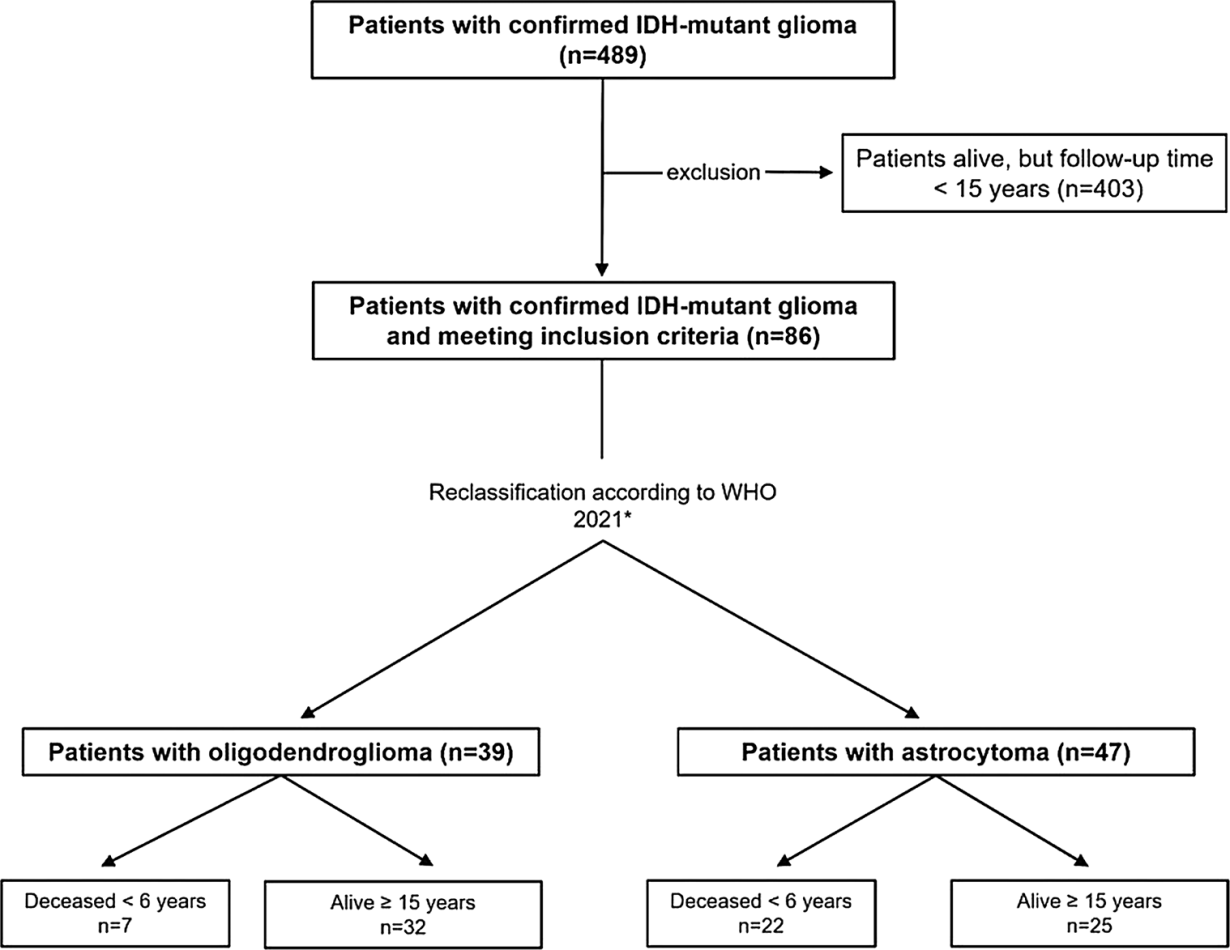
STROBE diagram. Patients with IDH-mutant gliomas were identified and included if death had been reported within 6 years after diagnosis or if the patient had survived for at least 15 years. Patients with shorter follow-up times were excluded. *Gliomas were classified according to WHO 2021 with the limitation of not having conducted CDKN2A/B analyses. *STROBE*,* The Strengthening the Reporting of Observational Studies in Epidemiology Statement*

The single-center, retrospective, case-control study was waived by the local ethical review committee of the LMU University Hospital, LMU Munich (project number 20–513 and project number 21–0612). The institutional database was screened for patients with IDH-mutant gliomas, confirmed by sequencing, and available 1p/19q-status between the years 1998 and 2023. Patients who had received a suspected magnetic resonance imaging (MRI)-based diagnosis of glioma or who had a tissue sample obtained before IDH and 1p/19q status was routinely determined but who then had molecular confirmation later in the clinical course were also retrospectively included (Fig. 1). Patients were included if they had undergone stereotactic biopsy or maximal safe resection [29, 30]. Clinical status was assessed retrospectively according to the Karnofsky Performance Status (KPS). Demographic data were collected. After baseline MRI, e.g., after completion of tumor-specific therapy, and in case of stable clinical status and imaging, routine intervals for MRI scans were 6 months in WHO grade 2 and 3 gliomas and 3 months in WHO grade 4 gliomas. For patients having undergone tumor resection, baseline MRI was defined as the first postoperative MRI scan. Data on PFS and OS were collected as outcome measures. For PFS, recommendations by the RANO group (Response Assessment in Neuro-Oncology) were followed and applied retrospectively [31]. Accordingly, either of the following observations determined progression: increase in tumor volume of 25% or more, any new lesion, or clinical deterioration not otherwise explicable. OS was defined as the time between date of first suspected diagnosis through MRI and date of death. Patients having died from conditions not related to tumor progression or therapy were not included in the study.

### Histopathology and molecular analyses

Histological samples underwent a thorough investigation and grading according to the WHO 2021 Classification of Tumours of the Central Nervous System by neuropathological consensus with the limitation of not having performed CDKN2A/B analyses. Accordingly, some CNS WHO grade 2 or 3 astrocytomas might have been classified as CNS WHO grade 4 in the WHO 2021 classification [17]. CNS WHO grade 2 and 3 oligodendrogliomas as well as CNS WHO grade 2, 3 and 4 astrocytomas were included. IDH1 and IDH2 mutations were confirmed both by immunohistochemistry and pyrosequencing of hotspot mutations in all patients. Quantitative microsatellite marker analysis was used to determine the 1p/19q status [32].

## Neuroimaging

Tumor location was assessed. Tumor volumes were measured by manual segmentation of T2 weighted images with 3-dimensional reconstruction. To this end, the commercially available Smartbrush Elements Software was utilized (Brainlab Elements Smartbrush, Munich, Germany). Contrast enhancement on T1-weighted sequences was assessed qualitatively. For differentiation between tumor infiltration and edema, criteria published by *Lasocki et al.* were applied by a neuroradiologist (C.T.) [33].

### Statistical analysis

The overall cohort was split according to survival and follow-up times in 2 groups: tumor-related death occurring < 6 years after diagnosis or survival of ≥ 15 years. Both groups were compared and then stratified according to glioma entity. All statistical testing was performed on GraphPad PRISM 9.4.1 software. If equal standard deviations could not be assumed, unpaired t-tests with Welch’s correction were used to compare continuous variables with normal distribution as confirmed by D’Agostino-Pearson test. Non-parametric data was analysed by Mann-Whitney U and Kruskal-Wallis tests. Chi-square test of independence or Fisher’s exact test whenever applicable were used for comparison of categorical variables. The proportional hazards assumption was tested by determining scaled Schoenfeld residuals. Hereafter, Cox proportional hazards models were used for univariate and multivariate testing. For categorical variables, the group with shorter OS time was used as a reference. Hazard ratios (HR) and 95% confidence intervals (95 CI) were calculated. Tumor grade was scaled continuously because it reflects a progression of severity and its correlation with outcome might be near linear. Accordingly, continuous scaling is more likely to detect associations and does not introduce arbitrary differences between each category. In multivariate analyses of OS times, only factors associated with survival times in univariate analyses were included. Survival analyses comprised Kaplan Meier estimates and Log-rank (Mantel-Cox) tests. The threshold to discriminate significant from non-significant results was set at a p-value ≤ 0.05.

## Results

### Study population and clinical parameters

The overall cohort consisted of 86 patients with 29 patients (34%) having died from tumor-related causes before reaching 6 years survival time and 57 patients (66%) living past 15 years after diagnosis (Fig. 1). Median follow-up of the survivors was 16.6 years (range 15-28.9). The median age of patients at diagnosis surviving < 6 years was 40 (range 14–70) and the mean initial KPS was 90 (range 20–100). In patients surviving ≥ 15 years, median age was 38 years (range 14–65) and mean KPS was also 90 (range 60–100) (Table 1). Overall, 81 patients (94%) were subjected to tissue sampling by resection or biopsy within 3 months after the first suspicious MRI. The female: male ratio was higher in the long-term survivor cohort (1.6:1 versus 0.6:1; *p* = 0.04). Within the cohort of patients who died before reaching the 6-year benchmark, 7 patients (24%) were diagnosed with oligodendroglioma and 22 patients (76%) were diagnosed with astrocytoma. In the long-term survival cohort, 32 patients (56%) were diagnosed with oligodendroglioma and 25 patients (44%) were diagnosed with astrocytoma (*p* < 0.01). Overall, wait-and-scan strategies had been initiated after diagnosis in 41 long-term survivors (72%) as opposed to 6 patients (21%) in the cohort of patients not surviving past 6 years. Within the cohort of patients initially monitored by means of wait-and-scan strategies, 41 (87%) had been diagnosed with CNS WHO grade 2 gliomas. Looking at patients having received any form of tumor treatment, significantly more patients with astrocytomas surviving less than 6 years as opposed to surviving at least 15 years had been treated with temozolomide alone (*p* < 0.01). In patients with oligodendrogliomas, a PCV therapy without radiotherapy had been given more often in the long-term survivor cohort by trend (*p* = 0.07). The number of long-term surviving patients having been treated with radiotherapy as opposed to chemotherapy alone did not differ significantly from the short-term survivor cohort (*p* = 0.34). In univariate analysis, regimens comprising radiotherapy were not associated with longer OS as compared to chemotherapy alone (HR 0.82; 95% CI 0.36–1.85; *p* = 0.63). Comparing patients having received radiotherapy versus chemotherapy alone showed no significant difference in the number of CNS grade 3 gliomas (*p* = 0.1) nor a higher number of contrast-enhancing tumors (*p* = 0.19). There were no statistically significant differences in the number of patients with oligodendroglioma versus astrocytoma in each cohort (*p* = 0.73). Patients treated with chemotherapy alone showed significantly larger tumors (89 cm^3^ versus 43 cm^3^; *p* = 0.04). Both oligodendrogliomas and astrocytomas were located most frequently in the frontal lobe. There was a trend towards more frequent frontal manifestations in patients who had an OS ≥ 15 years (*p* = 0.09). Patients who died < 6 years after diagnosis showed a higher proportion of WHO grade 3 or 4 gliomas (*p* < 0.01), had larger initial tumor volumes (*p* = 0.01), and contrast enhancement (*p* < 0.01). These significant differences persisted when looking at patients with astrocytoma and oligodendroglioma separately. In patients with oligodendroglioma, the primary tumor site was more frequently located on the left hemisphere or bilaterally in the short-term survivor cohort (*p* < 0.01). Of note, initial tumor volumenever exceeded 120 cm^3^ in long-term survivors. In short-term survivors, 7 patients showed initial tumor volumes of 200 cm^3^ or more. A comparison between patients having died within the first 15 years of diagnosis (*n* = 57) and patients surviving more than 15 years yielded similar differences than those described above (Suppl. Table S1).

**Table 1:** Patient and disease characteristics – survival < 6 years versus ≥ 15 years

Parameter	All patients, survival < 6 years (*n* = 29)	All patients, survival ≥ 15 years (*n* = 57)	*p*-value	Oligodendroglioma, survival < 6 years (*n* = 7)	Oligodendroglioma, survival ≥ 15 years (*n* = 32)	*p*-value	Astrocytoma, survival < 6 years (*n* = 22)	Astrocytoma, survival ≥ 15 years (*n* = 25)	*p*-value
**Age (years)**									
Median	40	38	*0.6*	48	39	*0.16*	37	38	*0.97*
Range	14–70	14–65		32–64	14–63		14–70	24–65	
**Sex**,** n (%)**									
Female	11 (38)	35 (61)	0.04*	2 (29)	19 (59)	*0.22*	9 (41)	16 (64)	*0.15*
Male	18 (62)	22 (39)		5 (71)	13 (41)		13 (59)	9 (36)	
**KPS**									
Mean	90	90	*0.22*	90	90	*0.04*	90	90	*0.8*
Range	20–100	60–100		20–90	60–100		70–100	80–90	
**Mode of tissue acquisition**,** n (%)**									
Biopsy	12 (41)	26 (46)	*0.82*	1 (14)	19 (59)	*0.04*	11 (50)	7 (28)	*0.14*
Resection	17 (59)	31 (54)		6 (86)	13 (41)		11 (50)	18 (72)	
**Initial management after surgery**,** n (%)**									
Wait-and-scan	6 (21)	41 (72)	*< 0.01*	3 (43)	23 (72)	*< 0.01*	3 (14)	18 (72)	*< 0.01*
Radiotherapy alone	5 (17)	6 (11)		2 (29)	2 (6)		3 (14)	4 (16)	
TMZ alone	13 (45)	1 (2)		2 (29)	0 (0)		11 (50)	1 (4)	
PC(V) alone	1 (4)	6 (11)		0 (0)	6 (19)		1 (5)	0 (0)	
Radiochemotherapy	4 (14)	3 (5)		0 (0)	1 (3)		4 (18)	2 (8)	
**Location**,** n (%)**									
Frontal	10 (35)	25 (44)	*0.09*	2 (29)	15 (47)	*0.13*	8 (36)	10 (40)	*0.36*
Temporal	6 (21)	13 (23)		2 (29)	5 (16)		4 (18)	8 (32)	
Insular	4 (14)	9 (16)		0 (0)	6 (19)		4 (18)	3 (12)	
Parietal	3 (10)	9 (16)		2 (29)	6 (19)		1 (5)	3 (12)	
Occipital	2 (7)	0 (0)		0 (0)	0 (0)		2 (9)	0 (0)	
Midline	4 (14)	1 (2)		1 (14)	0 (0)		3 (14)	1 (4)	
**Laterality**,** n (%)**									
Left	9 (31)	24 (42)	*0.16*	3 (43)	15 (47)	*< 0.01*	6 (27)	9 (36)	*0.82*
Right	17 (59)	32 (56)		2 (29)	17 (53)		15 (68)	15 (60)	
Bilateral	3 (10)	1 (2)		2 (29)	0 (0)		1 (5)	1 (4)	
**T2 tumor volume (cm** ^**3**^ **)**									
Median	89	53	*0.01*	175	55	*0.03*	78	49	*0.04*
Range	1-342	13–118		33–342	13–96		1-310	13–118	
**Contrast enhancement**									
Yes	16 (55)	12 (21)	*< 0.01*	5 (71)	9 (28)	*0.03*	11 (50)	3 (12)	*< 0.01*
No	11 (38)	40 (70)		1 (14)	20 (63)		10 (46)	20 (80)	
No data	2 (7)	5 (9)		1 (14)	3 (9)		1 (5)	2 (8)	
**CNS WHO grade**									
2	12 (42)	53 (93)	*< 0.01*	4 (57)	32 (100)	*< 0.01*	8 (36)	21 (84)	*< 0.01*
3	14 (48)	3 (5)		3 (43)	0 (0)		11 (50)	3 (12)	
4	3 (10)	1 (2)		-	-		3 (14)	1 (4)	

KPS, Karnofsky Performance Status; RT, radiotherapy; TMZ, temozolomide; PC(V), procarbazine, CCNU and vincristine. Significant p-values are highlighted with asterisks*

### Progression-free and overall survival

In the entire cohort, 82 patients (95%) had documented tumor progression. Only 4 patients (3%), exclusively patients with oligodendroglioma, did not show tumor progression after a follow-up ≥ 15 years. PFS was longer in patients surviving ≥ 15 years (in months, 90 versus 22, *p* < 0.01). Median survival in patients who lived < 6 years was 47 months and was not reached in patients who lived ≥ 15 years (*p* < 0.01). Overall, 34 patient deaths were documented. Only 5 events occurred ≥ 15 years after diagnosis. There were no non-tumor-related deaths in the entire cohort.

### Uni- and multivariate analyses

Univariate analyses were conducted for the entire cohort and included the following parameters: age and KPS at diagnosis, initial T2 tumor volume, contrast enhancement on MRI, tumor entity, CNS WHO grade, mode of tissue acquisition (biopsy versus resection), and therapeutic strategy (wait-and-scan versus tumor-specific therapy). Of these variables, a higher KPS, a lower CNS WHO grade, diagnosis of oligodendroglioma rather than astrocytoma, smaller T2 volumes, lack of contrast enhancement and wait-and-scan strategy were associated with longer OS (Table 2). The cohort was split according to tumor entity. In univariate analyses for oligodendroglioma, the following parameters were associated with longer OS: higher KPS at diagnosis, smaller T2 tumor volume and biopsy rather than tumor resection) (Table 3). Of these parameters, only T2 tumor volume persisted in multivariate analyses (HR 1.02; 95% CI 1.01–1.05; *p* = 0.04) (Table 4). In patients with astrocytoma, longer OS was associated with lower WHO grade, no contrast enhancement, and wait-and-scan strategies rather than early tumor-specific therapy (Table 5). In multivariate analyses, contrast enhancement (reference: contrast enhancement; HR 0.38; 95% CI 0.15–0.94; *p* = 0.04) and wait-and-scan strategies after tissue sampling rather than therapy initiation (reference: wait-and-scan; HR 5.75; 95% CI 1.66–26.61; *p* = 0.01) were associated with longer OS (Table 6).

**Table 2:** Univariate analyses for factors potentially related with overall survival

Factor	OS		
	HR	95% CI	*p*-value
Age^x^	1.01	0.98–1.04	*0.62*
KPS^x^	0.96	0.93-1.0	0.01*
Entity (oligodendroglioma versus astrocytoma)	2.48	1.19–5.65	0.02*
WHO grade (2/3)^x^	3.58	2.19–5.62	< 0.01*
T2 tumor volume^x^	1.01	1.00-1.01	< 0.01*
CE (yes/no)	0.35	0.16–0.73	< 0.01*
Mode of tissue acquisition (resection/biopsy)	1.21	0.61–2.51	*0.59*
Wait-and-scan versus therapy (radiotherapy and/or chemotherapy)	0.27	0.12–0.56	< 0.01*

KPS, Karnofsky Performance Status; WHO, World Health Organisation; CE, contrast-enhancement on MRI; OS, overall survival. Initial therapy comprised wait-and-scan strategies, temozolomide monotherapy, PCV chemotherapy, radiotherapy and radiochemotherapy. ^x^ Continuously scaled. Significant p-values are highlighted with asterisks*

**Table 3:** Univariate analyses for factors potentially related with overall survival in patients with oligodendroglioma

Factor	OS		
	HR	95% CI	*p*-value
Age^x^	1.02	0.97–1.08	*0.49*
KPS^x^	0.95	0.92-1.0	0.01*
WHO grade (2/3)^x^	1.85E + 13	range too wide	*> 0.99*
T2 tumor volume^x^	1.02	1.01–1.04	0.02*
CE (yes/no)	0.15	0.01–0.91	*0.08*
Mode of tissue acquisition (resection/biopsy)	10.85	10.9-201.6	0.03*
Wait-and-scan versus therapy (radiotherapy and/or chemotherapy)	1.77	0.42–7.49	*0.42*

KPS, Karnofsky Performance Status; WHO, World Health Organisation; CE, contrast-enhancement on MRI; OS, overall survival. Initial therapy comprised wait-and-scan strategies, temozolomide monotherapy, PCV chemotherapy, radiotherapy and radiochemotherapy. ^x^ Continuously scaled. Significant p-values are highlighted with asterisks*

**Table 4:** Multivariate analyses for factors potentially related with overall survival in patients with oligodendroglioma

Factor	OS		
	HR	95% CI	*p*-value
KPS^x^	0.66	0.28–0.99	*0.09*
T2 tumor volume^x^	1.02	1.01–1.05	0.04*
Mode of tissue acquisition (biopsy/resection)	range too wide	range too wide	*> 0.99*

KPS, Karnofsky Performance Status; OS, overall survival. ^x^ Continuously scaled. Significant p-values are highlighted with asterisks*

**Table 5:** Univariate analyses for factors potentially related with overall survival in patients with astrocytoma

Factor	OS		
	HR	95% CI	*p*-value
Age^x^	1.01	0.97–1.05	*0.71*
KPS^x^	0.96	0.88–1.07	*0.4*
WHO grade (2/3/4)^x^	2.67	1.53–4.54	< 0.01*
T2 tumor volume^x^	1.00	1.0-1.01	*0.18*
CE (yes/no)	0.27	0.11–0.65	< 0.01*
Mode of tissue acquisition (resection/biopsy)	2.19	0.95–5.08	*0.06*
Wait-and-scan versus therapy (radiotherapy and/or chemotherapy)	7.05	2.58–24.8	< 0.01*

KPS, Karnofsky Performance Status; WHO, World Health Organisation; CE, contrast-enhancement on MRI; OS, overall survival. Initial therapy comprised wait-and-scan strategies, temozolomide monotherapy, PCV chemotherapy, radiotherapy and radiochemotherapy. ^x^ Continuously scaled. Significant p-values are highlighted with asterisks*

**Table 6:** Multivariate analyses for factors potentially related with overall survival in patients with astrocytoma

Factor	OS		
	HR	95% CI	*p*-value
WHO grade (2/3/4)^x^	1.59	0.78–3.11	*0.19*
CE (yes/no)	0.38	0.15–0.94	0.04*
Wait-and-scan versus therapy (radiotherapy and/or chemotherapy)	5.75	1.66–26.61	0.01*

WHO, World Health Organisation; CE, contrast-enhancement on MRI; OS, overall survival. Initial therapy comprised wait-and-scan strategies, temozolomide monotherapy, PCV chemotherapy, radiotherapy and radiochemotherapy. ^x^ Continuously scaled. Significant p-values are highlighted with asterisks*

## Discussion

Risk factor assessment for patients with IDH-mutant gliomas is important for determination of timing and modality of tumor-specific therapy. To investigate prognostic factors, we compared a comparably large cohort of long-term survivors that were alive at least 15 years after diagnosis with patients who had died due to their underlying tumor disease within 6 years of initial diagnosis. Some patients were followed-up for more than 25 years. We report an association of WHO grade 2 histology (I), absence of initial contrast enhancement on MRI (II) and small initial tumor volume (III) with long-term survival (Table 1). There was no evidence for an association between age and survival, contrasting with the strong prognostic significance of age in glioblastoma [34–36]. 72% of the long-term survivors in our cohort had not received tumor-specific therapy after initial surgery and still showed a survival of ≥ 15 years. In multivariate analyses and stratification according to tumor entity (oligodendroglioma and astrocytoma), initial T2 tumor volume persisted as a prognostic marker for survival in patients with oligodendroglioma. Overall, only short-term survivors had tumors larger than 120 cm^3^ on initial MRI, underlining the potential prognostic importance of tumor burden. In patients with astrocytoma, lack of contrast enhancement and wait-and-scan strategies were associated with prolonged survival. This suggests that tumor size and contrast enhancement are strong prognostic factors, and that wait-and-scan therapy can yield excellent long-term outcome in some patients.

The standard of care for IDH-mutant gliomas is determined by large, prospective clinical trials comparing radiotherapy, chemotherapy and radiochemotherapy. These trials indicated that a sequential therapy, starting with radiotherapy and followed by chemotherapy, is most effective with regards to progression-free and overall survival, although this carries the risk of long-term adverse effects [5, 8, 19, 22–24, 37–39]. A potential consequence of these studies could be overtreatment and toxicity in some patients who might have shown a benign clinical course with less invasive strategies. Only a subset of patients with gross total resection, CNS WHO grade 2 histology or young patient age currently qualify for wait-and-scan strategies [14, 19]. For this subgroup, data is scarce. Prospective studies are difficult to conduct because of low incidence rates and long survival times. Already published studies need to be reevaluated considering the substantial changes in glioma definitions introduced by the WHO over the last decades. Not seldomly, prospective studies of these relatively rare gliomas need to be discontinued or adjusted, e.g., the IWOT trial (NCT03763422) that investigated wait-and-scan strategies or the CODEL trial (NCT00887146) that initially aimed at comparing radiotherapy alone with temozolomide alone or a combination therapy for WHO grade 3 oligodendroglioma. Early versus delayed treatment after surgery has shown improved PFS, but not OS [19, 40]. Large retrospective studies demonstrate that some patients survive for decades despite conservative management strategies [4, 9, 10]. Determining factors that have an impact on survival in patients with IDH-mutant gliomas is important to identify subgroups of patients who might be eligible for less aggressive initial treatment regimens. In that regard, the demonstration of prolonged progression-free survival in patients treated with the IDH inhibitor vorasidenib provides another approach to delay the use of radiochemotherapy in select patient populations [38].

Patients with oligodendroglioma are generally assumed to show a more favorable clinical course than those with IDH-mutant astrocytoma, which is reflected by the low fatality rate of 9 of 39 patients (23%) with oligodendroglioma versus 25 of 47 patients (53%) with astrocytoma in our dataset and substantiated by univariate analysis (Table 2) [8, 20, 21].

The major limitation of this study is its retrospective design and the low number of events in the oligodendroglioma cohort, which renders multivariate analyses difficult to interpret. A substantial selection bias regarding initial treatment strategies must be taken into account. The study justifies the conclusion that some patients show excellent long-term outcomes with initial wait-and-scan strategies, even without tumor resection, but one cannot deduce a non-inferiority to, or superiority over treatment-specific therapy, because these patients might shown an even better long-term survival with radiochemotherapy. Our findings merely suggest that a subset of patients might not require immediate treatment. It is of utmost importance to identify these patients and to this end, refined risk factor assessment is required. A head-to-head comparison between treatment regimens comprising radiotherapy versus chemotherapy alone (temozolomide or PCV) did not show any difference in efficacy. However, this comparison might be prone to a selection bias and might be questionable regarding the high efficacy of PCV in oligodendroglioma as opposed to the limited efficacy of temozolomide alone in astrocytoma patients. In our cohort, only four short-term survivor patients (18%) with astrocytoma received radiochemotherapy and many received temozolomide alone. This might have represented undertreatment in these patients, considering that the effect of temozolomide alone is controversial in this population [9, 27, 28]. Of note, many patients in this study had been treated before publication of landmark studies on lower grade gliomas. Current guidelines have changed drastically. Chemotherapy alone, a strategy that had been pursued in many patients included in this study initially, nowadays might only be indicated in individual patients, e.g. a young patient with a large, non-resectable tumor. A further limitation is the lack of data on postoperative, residual tumor volume which might be an important risk factors for shorter PFS and OS [11]. Interpretation of wait-and-scan strategies is limited by the fact that 87% of patients not receiving tumor-specific therapy at first diagnosis were diagnosed with WHO grade 2 gliomas. These patients are expected to show a more favorable clinical course. Ultimately, the prognostic relevance of WHO grade in patients with oligodendroglioma cannot be determined yet based on our study, as only 3 patients with CNS WHO grade 3 oligodendroglioma were included.

In summary, the results demonstrate that a subset of patients with IDH-mutant glioma shows excellent survival times with initial wait-and-scan strategy. When evaluating therapy strategies in these tumors, ruling out CDKN2A/B deletions might be indicated, as wait-and-scan strategies are not warranted in higher grade glioma. Contrast enhancement and initial tumor size might be especially important in risk factor assessment and these factors should be included in the decision making for postoperative treatment. Age at diagnosis might not be an important predictor of long-term survival.

## Electronic supplementary material

Below is the link to the electronic supplementary material.

Supplementary Material 1

## Data Availability

No datasets were generated or analysed during the current study.

## Declarations

### Additional declarations for articles in life science journals that report the results of studies involving humans and/or animals

The present study was conducted retrospectively.
