# Supplementary material for: Determinants of long-term survival in patients with IDH-mutant gliomas
Source: J Neurooncol. 2024 Sep 24;170(3):655–64. doi: 10.1007/s11060-024-04826-9 (PMC11614945; doi:10.1007/s11060-024-04826-9)
Supplement: Supplementary file 1 — Supplementary Material 1 [file 11060_2024_4826_MOESM1_ESM.docx]

# **Supplementary Material**

Suppl. table 1. Patient and disease characteristics – survival ≤15 years versus >15 years

| **Parameter** | **All patients, survival <15 years**  **(n=57)** | **All patients, survival ≥15 years (n=57)** | ***p-value*** | **Oligodendroglioma, survival <15 years**  **(n=16)** | **Oligodendroglioma, survival ≥15 years**  **(n=32)** | ***p-value*** | **Astrocytoma,**  **survival**  **<15 years**  **(n=41)** | **Astrocytoma, survival**  **≥15 years**  **(n=25)** | ***p-value*** |
| --- | --- | --- | --- | --- | --- | --- | --- | --- | --- |
| **Age (years)** |  |  |  |  |  |  |  |  |  |
| Median | 39 | 38 | *0.84* | 43 | 39 | *0.23* | 37 | 38 | *0.79* |
| Range | 14-70 | 14-65 |  | 26-64 | 14-63 |  | 14-70 | 24-65 |  |
| **Sex, n (%)** |  |  |  |  |  |  |  |  |  |
| Female | 23 (40) | 35 (61) | ***0.04**** | 6 (38) | 19 (59) | *0.22* | 17 | 16 (64) | *0.13* |
| Male | 34 (60) | 22 (39) |  | 10 (63) | 13 (41) |  | 24 | 9 (36) |  |
| **KPS** |  |  |  |  |  |  |  |  |  |
| Mean | 90 | 90 | *0.13* | 90 | 90 | *0.19* | 90 | 90 | *0.39* |
| Range | 20-100 | 60-100 |  | 20-90 | 60-100 |  | 70-100 | 80-90 |  |
| **Mode of tissue acquisition, n (%)** |  |  |  |  |  |  |  |  |  |
| Biopsy | 23 (40) | 26 (46) | *0.71* | 4 (25) | 19 (59) | ***0.03**** | 19 (46) | 7 (28) | *0.2* |
| Resection | 34 (60) | 31 (54) |  | 12 (75) | 13 (41) |  | 22 (54) | 18 (72) |  |
| **Initial management after surgery, n (%)** |  |  |  |  |  |  |  |  |  |
| Wait-and-scan | 16 (28) | 41 (72) | ***<0.01**** | 7 (44) | 23 (72) | ***<0.01**** | 9 (22) | 18 (72) | ***<0.01**** |
| Radiotherapy alone | 12 (21) | 6 (11) |  | 3 (19) | 2 (6) |  | 9 (22) | 4 (16) |  |
| TMZ alone | 21 (37) | 1 (2) |  | 6 (38) | 0 (0) |  | 15 (37) | 1 (4) |  |
| PC(V) alone | 1 (2) | 6 (11) |  | 0 (0) | 6 (19) |  | 1 (2) | 0 (0) |  |
| Radiochemotherapy | 7 (12) | 3 (5) |  | 0 (0) | 1 (3) |  | 7 (17) | 2 (8) |  |
| **Location, n (%)** |  |  |  |  |  |  |  |  |  |
| Frontal | 23 (40) | 25 (44) | *0.18* | 7 (44) | 15 (47) | *0.47* | 16 (39) | 10 (40) | *0.62* |
| Temporal | 13 (23) | 13 (23) |  | 3 (19) | 5 (16) |  | 10 (24) | 8 (32) |  |
| Insular | 9 (16) | 9 (16) |  | 2 (13) | 6 (19) |  | 7 (17) | 3 (12) |  |
| Parietal | 4 (7) | 9 (16) |  | 2 (13) | 6 (19) |  | 2 (5) | 3 (12) |  |
| Occipital | 3 (5) | 0 (0) |  | 1 (6) | 0 (0) |  | 2 (5) | 0 (0) |  |
| Midline | 5 (9) | 1 (2) |  | 1 (6) | 0 (0) |  | 4 (10) | 1 (4) |  |
| **Laterality, n (%)** |  |  |  |  |  |  |  |  |  |
| Left | 23 (40) | 24 (42) | *0.24* | 9 (56) | 15 (47) | *0.07* | 14 (34) | 9 (36) | *0.3* |
| Right | 29 (51) | 32 (56) |  | 5 (31) | 17 (53) |  | 24 (59) | 15 (60) |  |
| Bilateral | 5 (9) | 1 (2) |  | 2 (13) | 0 (0) |  | 3 (7) | 1 (4) |  |
| **T2 tumor volume (cm^3^)** |  |  |  |  |  |  |  |  |  |
| Median | 96 | 53 | ***<0.01**** | 127 | 55 | ***0.02**** | 86 | 49 | ***0.03**** |
| Range | 1-342 | 13-118 |  | 33-342 | 13-96 |  | 1-310 | 13-118 |  |
| **Contrast enhancement** |  |  |  |  |  |  |  |  |  |
| Yes | 25 (44) | 13 (23) | ***0.02**** | 9 (56) | 9 (28) | ***0.05**** | 17 (42) | 3 (12) | ***0.01**** |
| No | 26 (46) | 39 (68) |  | 5 (31) | 20 (63) |  | 20 (49) | 20 (80) |  |
| No data | 6 (11) | 5 (9) |  | 2 (13) | 3 (9) |  | 4 (10) | 2 (8) |  |
| **CNS WHO grade** |  |  |  |  |  |  |  |  |  |
| 2 | 28 (49) | 53 (93) | ***<0.01*** | 11 (69) | 32 (100) | ***<0.01**** | 17 (42) | 21 (84) | ***<0.01**** |
| 3 | 25 (44) | 3 (5) |  | 5 (31) | 0 (0) |  | 20 (49) | 3 (12) |  |
| 4 | 4 (7) | 1 (2) |  | - | - |  | 4 (10) | 1 (4) |  |

*KPS, Karnofsky Performance Status; RT, radiotherapy; TMZ, temozolomide; PC(V), procarbazine, CCNU and vincristine. Significant p-values are highlighted with asterisks**
